# Synthesis and characterization of porous silicon as hydroxyapatite host matrix of biomedical applications

**DOI:** 10.1371/journal.pone.0173118

**Published:** 2017-03-14

**Authors:** A. Dussan, S. D. Bertel, S. F. Melo, F. Mesa

**Affiliations:** 1 Universidad Nacional de Colombia, Facultad de Ciencias, Departamento de Física, Grupo de Materiales Nanoestructutrados y sus Aplicaciones, Ciudad Universitaria, Bogotá, Colombia; 2 Universidad del Rosario, Facultad de Ciencias Naturales y Matemáticas, Grupo NanoTech, Bogotá, Colombia; Institute of Materials Science, GERMANY

## Abstract

In this work, porous-silicon samples were prepared by electrochemical etching on p-type (B-doped) Silicon (Si) wafers. Hydrofluoric acid (HF)-ethanol (*C*_2_*H*_5_*OH*) [HF:Et] and Hydrofluoric acid (HF)-dimethylformamide (DMF-*C*_3_*H*_7_*NO*) [HF:DMF] solution concentrations were varied between [1:2]—[1:3] and [1:7]—[1:9], respectively. Effects of synthesis parameters, like current density, solution concentrations, reaction time, on morphological properties were studied by scanning electron microscopy (SEM) and atomic force microscopy (AFM) measurements. Pore sizes varying from 20 nm to micrometers were obtained for long reaction times and [HF:Et] [1:2] concentrations; while pore sizes in the same order were observed for [HF:DMF] [1:7], but for shorter reaction time. Greater surface uniformity and pore distribution was obtained for a current density of around 8 mA/cm^2^ using solutions with DMF. A correlation between reflectance measurements and pore size is presented. The porous-silicon samples were used as substrate for hydroxyapatite growth by sol-gel method. X-ray diffraction (XRD) and SEM were used to characterize the layers grown. It was found that the layer topography obtained on PS samples was characterized by the evidence of Hydroxyapatite in the inter-pore regions and over the surface.

## Introduction

Porous silicon (PS), since its discovery [1–4], has had huge development for various applications, like gas sensors [5], solar cells [6, 7], Bragg reflectors [8], biosensors [9–12], photocatalysis [13], tissue engineering [14], and cell growth [15–18], among others. Particularly, the last two applications have attracted researcher interest in studying the properties of PS as a host matrix Hydroxyapatite (HA) and its implementation in the development of processes leading to bone regeneration. Sygnatowicz and Tiwari, have reported HA studies for biomedical application [19].

Porous silicon is manufactured through electrochemical etching [20–22], using hydrofluoric acid (HF) and organic electrolytes, like sulfuric acid (*H*_2_*SO*_4_), hydrogen peroxide (*H*_2_*S*_2_), and ethanol (*C*_2_*H*_5_*O*), etc. [23–25]. The architecture in the formation of pores and their correlation with the synthesis parameters has been extensively evidenced for use in the development of scaffold-type structures [26, 27]; however, understanding the kinetics of pore formation and the influence of the synthesis parameters using Dimethylformamide (DMF- *C*_3_*H*_7_*NO*) as electrolyte is still unclear.

In this work PS samples were obtained through electrochemical etching from [HF:Et] (Hydrofluoric acid—Ethanol) and [HF:DMF] (Hydrofluoric acid—Dimethylformamide) solutions, varying the synthesis parameters such as: concentration, current density (J), and reaction time (t); the temperature (T) during the synthesis process no was varied (room temperature). Morphology and surface topography were studied using SEM and AFM measurements, identifying a pore-size variation between 20 and 170 nm using both solutions (Et and DMF). A correlation between synthesis parameters and I-V curves as a function of the formation of the porous surface and the influence of the electrolyte solution is presented.

An important contribution in this work is the comparative study between Et and DMF solutions for the porous formation on silicon, and their biomedical application in the grown of Hydroxyapatite.

## Experimental

### Material synthesis

Porous silicon (PS) samples were obtained via electrochemical etching using P-type silicon wafers (Boron-doped) with resistivity between 0.001 and 0.005 Ωcm, (*100*) crystallographic orientation, and thickness of 525 ±25*μm*. From Hydrofluoric acid (HF)-ethanol [HF:Et] and Hydrofluoric acid (HF)-dimethylformamide (DMF) [HF:DMF] solutions (40% HF Merck KGaA, Et Absolute for analysis Merck KGaA, 99,96% DMF J.T.Baker), with concentrations between [1:2] and [1:9] for both cases, current density *J* and *t* synthesis parameters were varied. For concentrations of [HF:Et], *J* was varied between 50 and 150 mA/cm^2^ and the reaction time was between 1 and 20 min. When used as an electrolyte solution in DMF, *J* varied between 2 and 8 mA/cm^2^, and *t* varied between 20 and 120 min. In all cases, the samples were deposited at room temperature, with a pH of 7–8 and with an area of contact about 0.78 cm^2^. The parameter values (J, t) reported here, were obtained after the many samples prepared (around 40 controlled trial); specifically, the J range was obtained form of cyclic voltammetry (CV) measurements. I (V) characteristic was obtained between -10 and 10 V and scan rate of 100 mV/s.

In order to carry out the tests of PS samples as Hydroxyapatite (HA) host matrix, calcium hydroxyapatite *Ca*_10_(*PO*_4_)_6_(*OH*)_2_ was synthesized by using the sol-gel method. The HA synthesis process was performed using Calcium nitrate tetrahydrate (*Ca*(*NO*_3_)_2−4_*H*_2_*O*, Merck), and Phosphorus pentoxide (*P*_2_*O*_5_, J.T Baker). These reactants were combined maintaining a stoichiometric ratio of 1.67. From of powder state, it was solubilize in deionized water and using spin coating process was deposited on PS samples. Sol gel method has been reported using other stoichiometric ratio [28–30].

### Characterizations

The PS samples were characterized by X-ray diffraction (XRD) (Philips X'Pert diffractometer) using PANalytical Pro, equipped with a source of Cu-Kα: 1.540598 Å, voltage at 40 kV, current at 40 mA, and X'Celerator detector. The software used to collate the results was the X'Pert HighScore Plus by Rietveld refinement and simulation of crystal structures complemented by the Jmol software. Scanning electron microscopy (SEM) and Energy Dispersive X-ray (EDX) (JEOL JSM-6490LV) measurements were obtained with 3.0-nm resolution, magnification × 5~300.000 and 0.3~30 kV accelerating voltage under high vacuum (~10^−6^ mbar). Atomic force microscopy (AFM) measurements were carried out using a Asylum Research MFP 3D system in tapping mode. Spectrophotometer Cary 5000 of UV-VIS-NIR at atmospheric pressure and room temperature was used for to obtain the reflectance measurements as a function of wavelength. The cyclic voltammetry experiments (CV) were carried out through a potentiostat (Princeton Applied Research, PARSTAT 2263) using a Electrochemistry PowerSuite software for data collected. This system has 3-electrode cell with a platinum wire as the counter electrode, a Si/Al substrate as the working electrode, and an Ag/AgCl (3 MNaCl) reference electrode; a analog system reported by Bhattacharyya [31].

## Results and discussion

**Fig 1:**
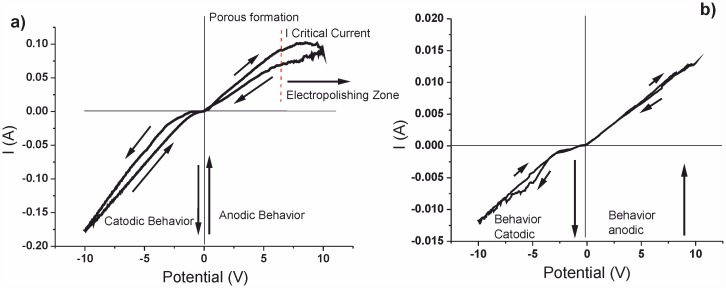
Voltammograms of PS samples between -10 and 10 V and scan rate of 100 mV/s: a) PS [HF:Et] [1:2] and b) [HF:DMF] [1:7].

From the range of the anodization parameters considered, a study of the current (I) as a function of potential (V) was performed. This characterization during anodizing processes gives information on the behavior of the solution during the reaction and identifies the value of I for which it is possible to differentiate the region where the pore formation process is carried out. Other region is known as electro-polished (I_critical_). Fig 1 shows cyclic voltammetry (CV) measurements between -10 V and 10 V for Si:B samples with concentrations [HF:Et] [1:2] (Fig 1A) and [HF:DMF] [1:7] (Fig 1B).

The region for negative current values corresponds to the cathode region of the I-V curve. This region is dominated by hydrogen reduction processes and, therefore, is less sensitive to these types of ions within the aqueous solution. In this reaction, the release of hydrogen gas was evident as a result of water reduction in the Si/HF interface. This was observed with the formation of bubbles in the solution during the experiment. On the other hand, in the anodic region of the curve, the Si dissolves in the HF electrolyte. The polished surface can have roughness, electro polish, or undergo nucleation of pores producing the porous layer. This is denoted by the critical point in the curve indicated in Fig 1 with dotted lines.

Table 1 shows the values obtained of pore size diameter of PS samples after the electrochemical processes. From PSI AP-0100 software and SEM measurements were obtained average pore sizes. It was observed that, with Et concentrations of [1:7] and t ≥ 60 min the pore formation was major in comparasion with [1:9] concentration. However, using [1:2] concentration DMF there are pores when J values are between 100 and 150 mA/cm^2^.

**Table 1:** Values obtained for the pore size of PS samples grown by the electrochemical anodization method.

Sample	J (mA/cm^2^)	t (min)	Concentration HF—Surfactant	Pore size diameter (∅)
m1a	2	30	[1:9]	Not appreciable
m1b	2	60	[1:9]	2 nm<∅≤50 nm
m1c	2	120	[1:9]	Cracks ≤25 μm
m2a	4	30	[1:9]	Not appreciable
m2b	4	60	[1:9]	Cracks ≤ 1,5 μm
m2c	4	120	[1:9]	Cracks ≤ 8 μm
m3a	8	30	[1:9]	Cracks < 4 μm
m3b	8	60	[1:9]	≤ 1,7 μm
m3c	8	120	[1:9]	Not appreciable
m4b	2	60	[1:7]	25 nm<∅≤1,32 μm
m4c	2	120	[1:7]	Electropolished, Cracks < 5 μm
m5a	4	30	[1:7]	Cracks < 10 μm
m5b	4	60	[1:7]	≤ 70 nm
m6b	8	60	[1:7]	< 3 μm
m6c	8	120	[1:7]	< 2,2 μm
M9Et	100	1	[1:2]	≤ 10 μm
M1Et	50	1	[1:2]	Not appreciable
M17Et	150	10	[1:2]	≤ 170 nm
M19Et	180	20	[1:2]	Cracks < 5 μm

**Fig 2:**
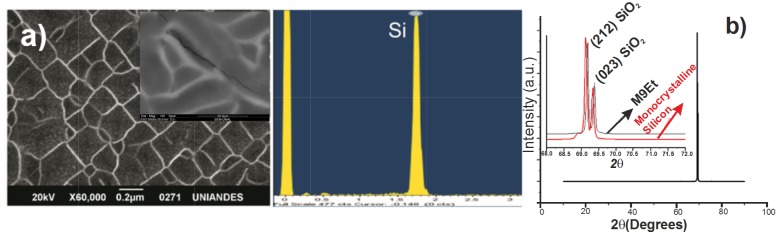
PS sample with [HF:Et] solution at [1:2]: a) SEM micrograph and EDX spectrum, b) diffraction pattern. The synthesis parameters were *J* = 150 mA/cm^2^ and *t* = 10 min. The inset correspond to SEM image of PS obtained for a reaction time major (t = 20 min and *J* = 180 mA/cm^2^).

In order to have detailed information of the samples, XRD, SEM and EDX measurements were carried out (Fig 2). In this case, the [HF:Et] concentration was [1:2] with *J* = 150 mA/cm^2^ and *t* = 10 min. A SEM micrograph of PS sample [HF:Et] with same concentration, but a reaction time of 20 min and *J* = 180 mA/cm^2^ is showed in the inset of Fig 2A; this image has 5000x magnification and 30.0 KV

Fig 2A reveals the presence of regions with pore of sizes averaging around 170±3 *nm* and some others with sizes greater; this last can be associated to the deterioration of walls in the formation of the pore by effect of the reaction time and, later, breaking of the same generating a major size (> 500 nm); likewise, the diffraction measurement shown in Fig 2B shows the porous condition of the sample with evidence of crystalline peak shift 2*θ* = 69.3 with values 2*θ* = 69.5, compared to the silicon wafer pattern used as substrate. This shift is associated to the presence of pores or texture of the material, that present after the etching processes [32]. Values of J > 100 mA/cm^2^ and *t* < 15 min allow for uniform pore formation on the surface when using the solution containing ethanol; however, for higher values of J (*J* = 180 mA/cm^2^) and reaction time (t = 20 min) a cracking of silicon surface is observed (Inset Fig 2A), with greater evidence of SiO_2_ phases in the nanostructure. This characteristic of cracking or electropolishing condition has been reported by H. Ohji et al [33].

**Fig 3:**
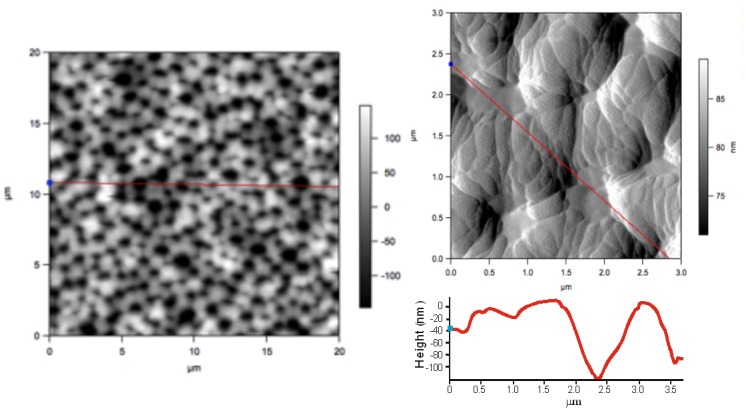
AFM image of PS sample with synthesis parameters *t* = 60 min, *J* = 2 mA/cm^2^ and [HF:DMF] at [1:7] concentration: a) AFM image in 2D, b) Three-dimensional image of the pore.

Fig 3 shows AFM images of the PS under solution with DMF electrolyte at [1:7] concentration. Synthesis parameters, J and t, were 2 mA/cm^2^ and 60 min, respectively.

Samples prepared with different [HF:DMF] concentrations exhibit greater pore formation uniformity for reaction times varying between 10 and 120 min (Fig 3A), compared to the samples prepared by using ethanol (*t* < 20 min). In Fig 3B a magnification of the PS surface is presented, with pore sizes of 1.4 ± 0.1*μm* at the top and depth in the order of 120 ± 10 *nm*. Using DMF significantly favors pore formation when reaction times are high, compared to times assigned when using ethanol.

**Fig 4:**
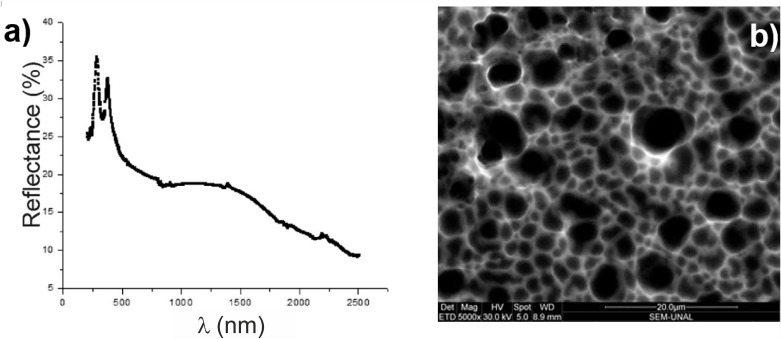
a) Reflectance spectrum as a function of wavelength of the PS sample made with *J* = 8 mA/cm^2^, concentration [HF: DMF] [1: 7] and *t* = 120 min. b) SEM micrograph of the porous surface.

Fig 4 shows the reflectance spectrum (R) and SEM image of the PS sample, using [HF:DMF] solution at [1:7] and current density *J* = 8 mA/cm^2^ with reaction time of 120 min. Fig 4A can be divided into two regions: a region where R is characterized by the presence of two peaks in the spectrum for the range of short wavelengths (λ) (between 200 and 500 nm, UV region) and directly associated to the sample’s porous condition [34,35], where reflectivity is described by the Fresnel relation through the complex refractive index; while increased absorbance is observed for the *λ* ≥ 2000 *nm* region (IR region), linked to light-scattering factors and high surface roughness values of the material (Fig 4B).

**Fig 5:**
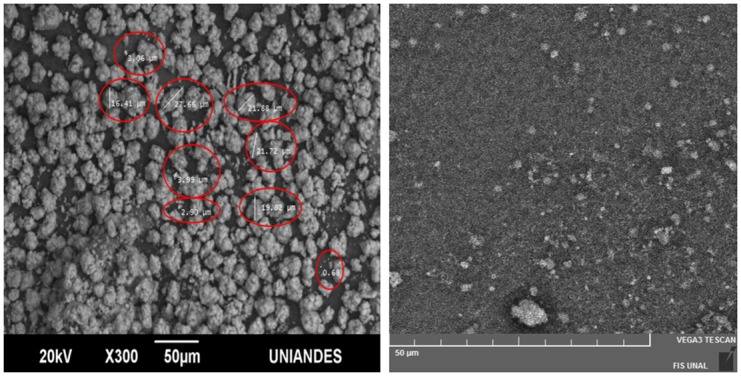
a) SEM micrograph of HA synthesized by the sol-gel method, showing regions with red circles where the grain size of HA was measured. b) SEM micrograph of the sample PS + HA. PS sample was synthesized with concentrations of ethanol [1: 2], *J* = 100 mA/cm^2^ and synthesis time 1 min.

After obtaining the samples of PS, HA was deposited on the surface of the samples, after having been subjected to screening processes in order to obtain HA sizes of the order of microns. Fig 5 shows SEM micrographs of HA synthesized by sol-gel method [28] with grain sizes varying between 0.68 μm and 27.66 μm (red circles highlighted in Fig 5A). Considering that the HA synthesized has different grain sizes, sieving processes were carried out with meshes that allowed selection of HA crystals smaller than 1 μm for provision on PS samples by the spin coating method. Fig 5B shows the PS sample with HA on the surface. A HA cluster was observed isolated, and it can be associated to the formation of the big grains in the spin-coating stage.

Fig 5B shows an even distribution of HA on the PS sample, allowing its application on the growth of osteoblasts in bone regeneration processes. However, in other applications the HA has been considered for their antibacterial properties [36].

## Conclusion

In this work, porous-silicon samples were prepared by using ethanol and DMF as electrolyte at different concentrations in the solution (S1 Table). Pore formation was favored in with ethanol for large current densities and shorter times at 20 min, in contrast to the formation of the porous surface when DMF was used, obtained above 120 min and low current densities. Hydroxyapatite grain size < 1 *μm* were observed with a major probability of inclusion into PS matrix for applications in cell growth (S1–S5 Figs).

## Supporting information

S1 Fig Voltammograms of PS samples between -10 and 10 V and scan rate of 100 mV/s: a) PS [HF:Et] [1:2] and b) [HF:DMF] [1:7]. (TIF)

S2 Fig PS sample with [HF:Et] solution at [1:2]: a) SEM micrograph and EDX spectrum, b) diffraction pattern. The synthesis parameters were *J* = 150 mA/cm^2^ and *t* = 10 min. The inset correspond to SEM image of PS obtained for a reaction time major (t = 20 min and *J* = 180 mA/cm^2^).
(TIF)

S3 Fig AFM image of PS sample with synthesis parameters *t* = 60 min, *J* = 2 mA/cm^2^ and [HF:DMF] at [1:7] concentration: a) AFM image in 2D, b) Three-dimensional image of the pore. (TIF)

S4 Fig a) Reflectance spectrum as a function of wavelength of the PS sample made with *J* = 8mA/cm^2^, concentration [HF: DMF] [1: 7] and *t* = 120min. b) SEM micrograph of the porous surface.
(TIF)

S5 Fig a) SEM micrograph of HA synthesized by the sol-gel method, showing regions with red circles where the grain size of HA was measured. b) SEM micrograph of the sample PS + HA. PS sample was synthesized with concentrations of ethanol [1: 2], *J* = 100 mA/cm^2^ and synthesis time 1 min.
(TIF)

S1 Table Values obtained for the pore size of PS samples grown by the electrochemical anodization method. (DOCX)
