# Supplementary figures and images for: Synthesis and characterization of porous silicon as hydroxyapatite host matrix of biomedical applications
Source: PLoS One. 2017 Mar 14;12(3):e0173118. doi: 10.1371/journal.pone.0173118 (PMC5349455; doi:10.1371/journal.pone.0173118)

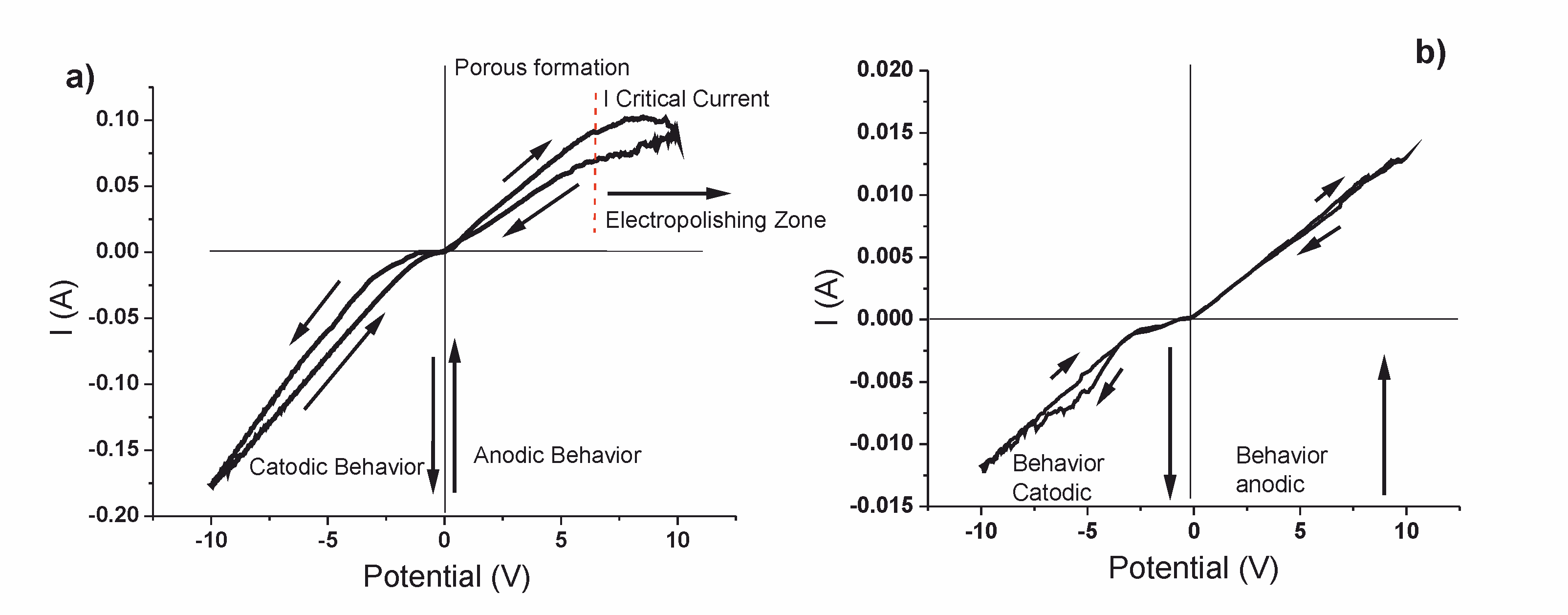

Supplement: S1 Fig — (TIF) [file pone.0173118.s001.tif]

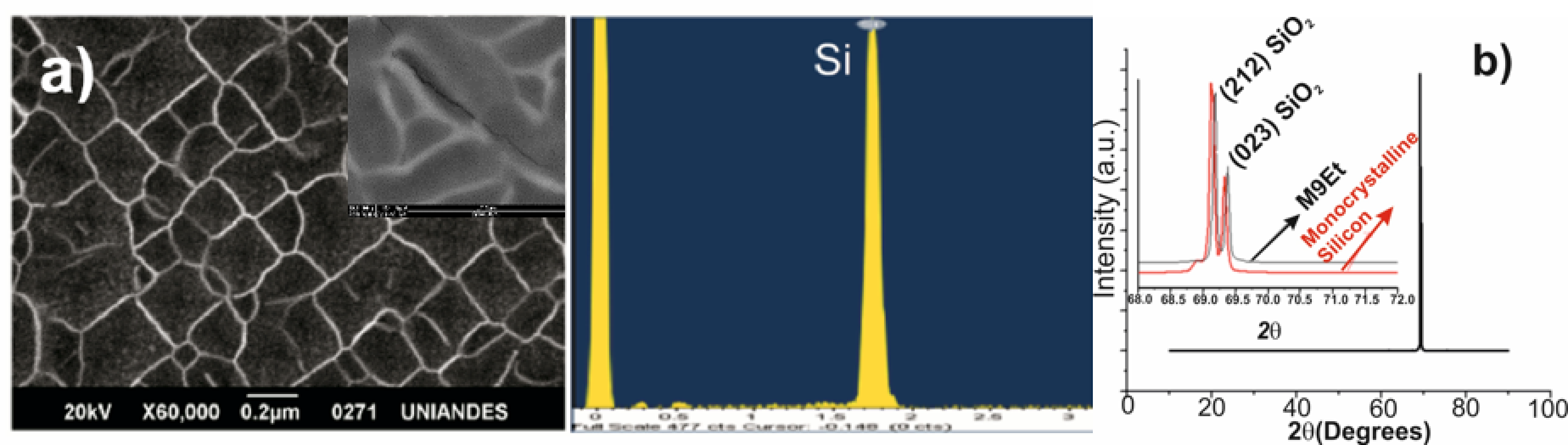

Supplement: S2 Fig — The synthesis parameters were J = 150 mA/cm2 and t = 10 min. The inset correspond to SEM image of PS obtained for a reaction time major (t = 20 min and J = 180 mA/cm2). (TIF) [file pone.0173118.s002.tif]

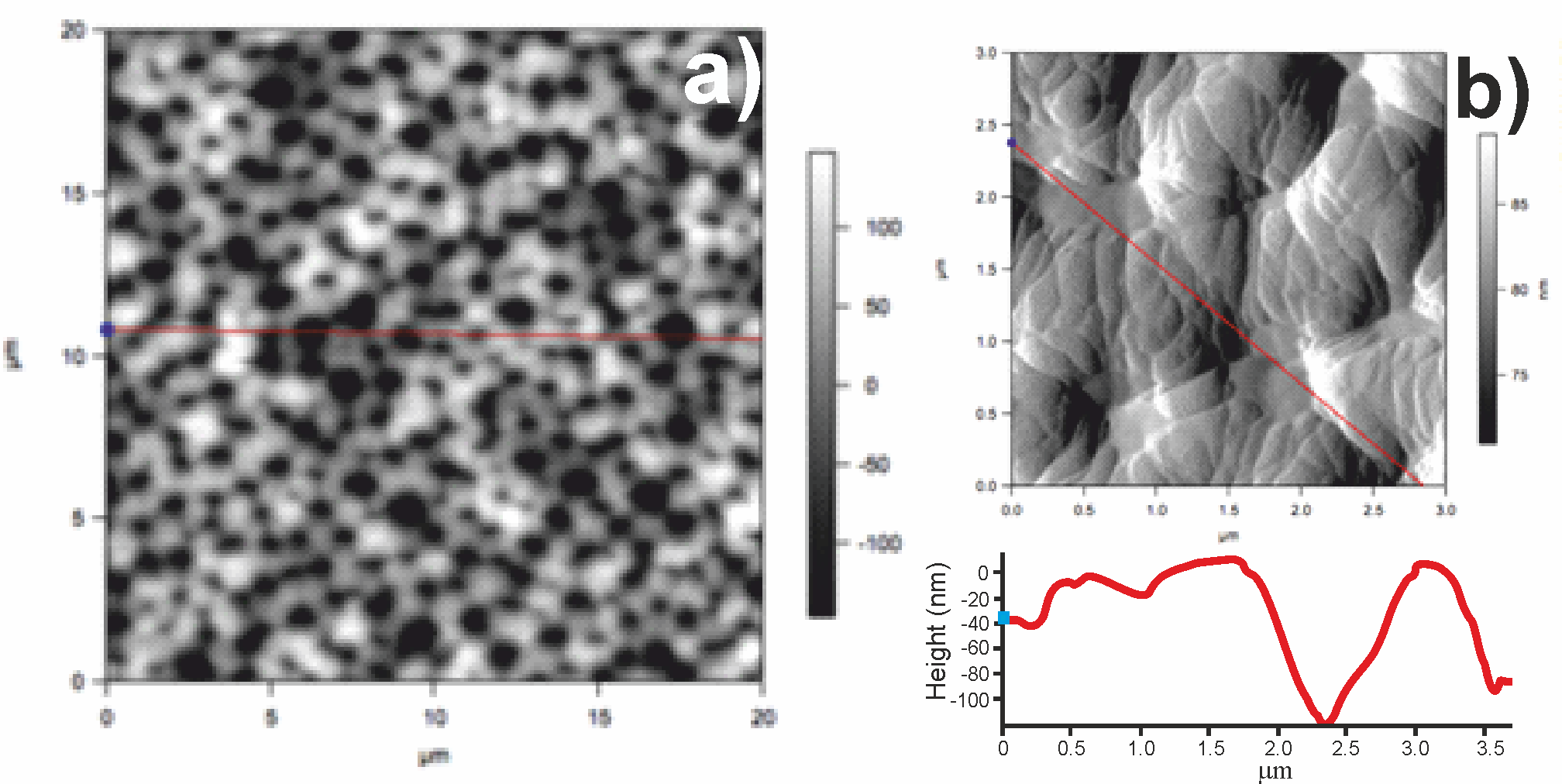

Supplement: S3 Fig — (TIF) [file pone.0173118.s003.tif]

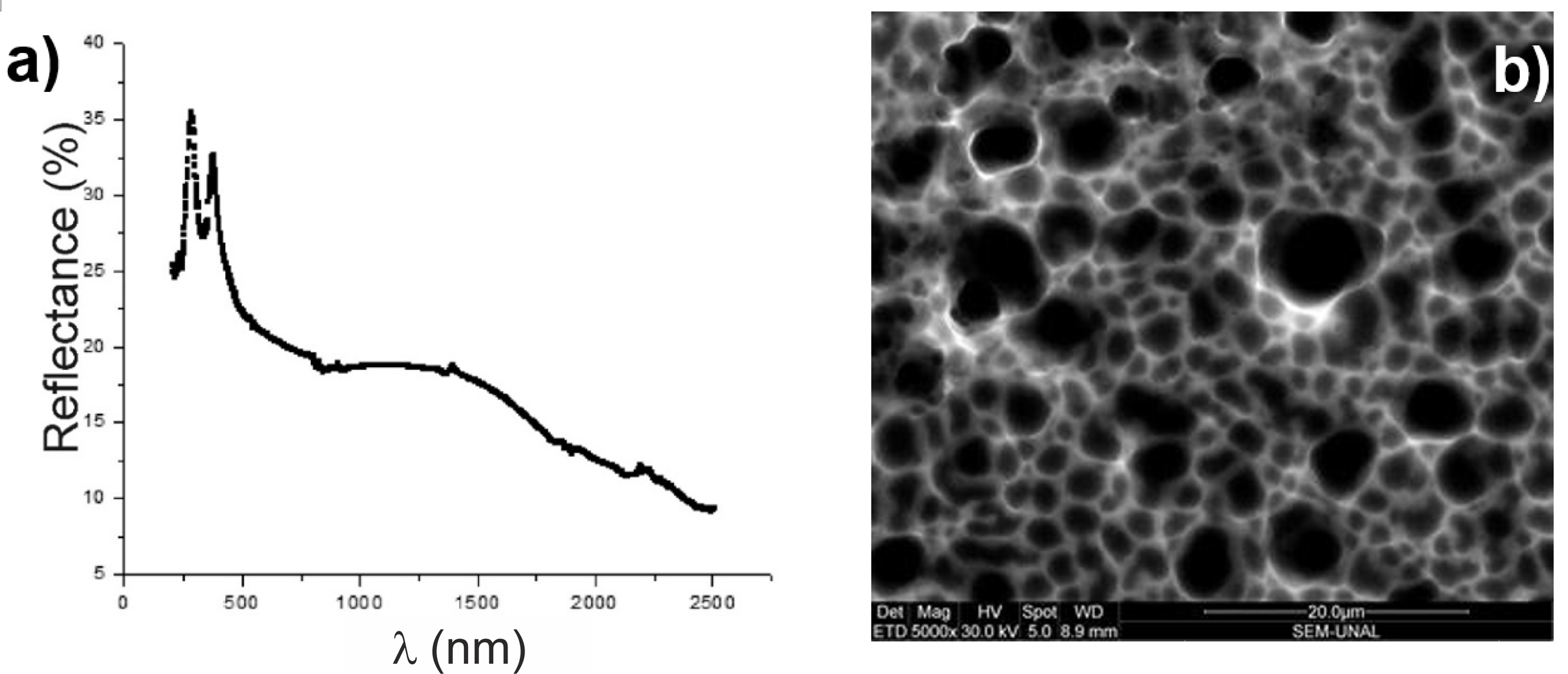

Supplement: S4 Fig — a) Reflectance spectrum as a function of wavelength of the PS sample made with J = 8mA/cm2, concentration [HF: DMF] [1: 7] and t = 120min. b) SEM micrograph of the porous surface. (TIF) [file pone.0173118.s004.tif]

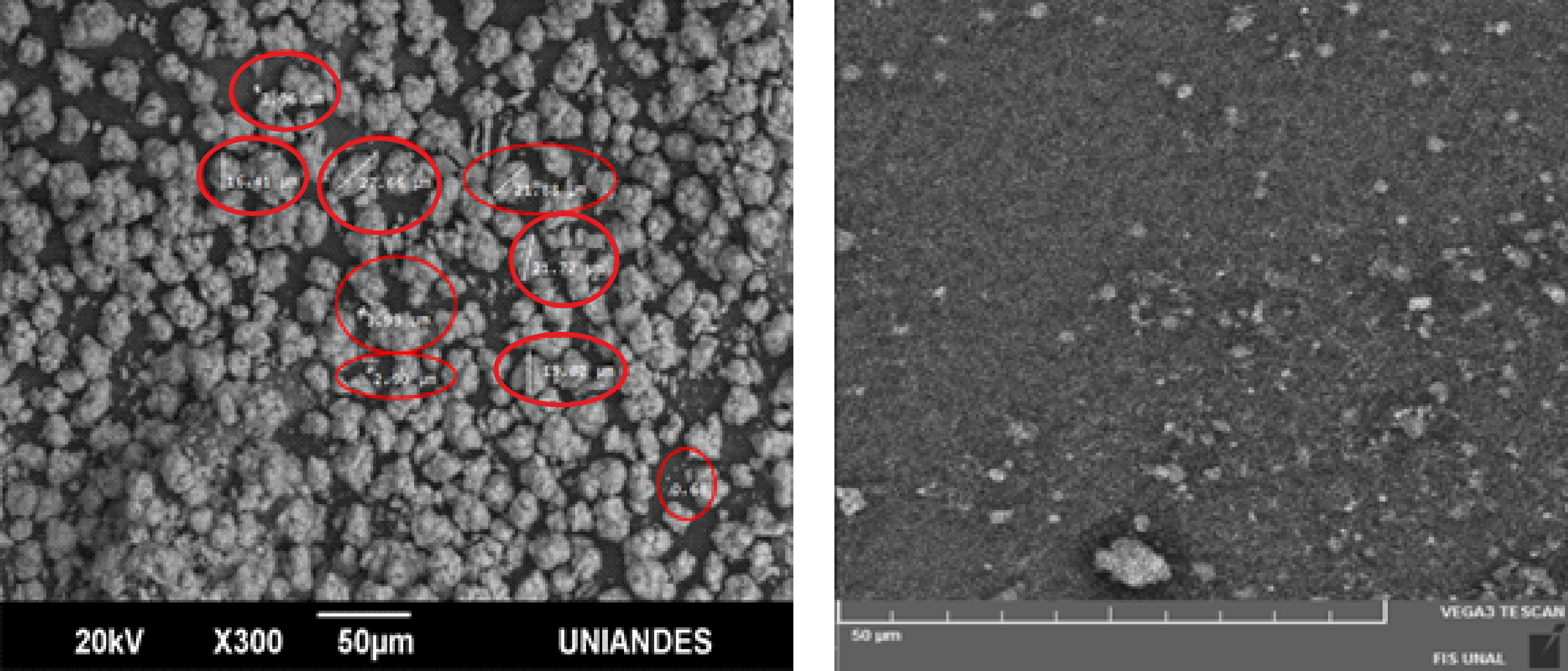

Supplement: S5 Fig — a) SEM micrograph of HA synthesized by the sol-gel method, showing regions with red circles where the grain size of HA was measured. b) SEM micrograph of the sample PS + HA. PS sample was synthesized with concentrations of ethanol [1: 2], J = 100 mA/cm2 and synthesis time 1 min. (TIF) [file pone.0173118.s005.tif]
