# Supplementary material for: Synthesis and characterization of porous silicon as hydroxyapatite host matrix of biomedical applications
Source: PLoS One. 2017 Mar 14;12(3):e0173118. doi: 10.1371/journal.pone.0173118 (PMC5349455; doi:10.1371/journal.pone.0173118)
Supplement: S1 Table — (DOCX) [file pone.0173118.s006.docx]

Table 1. Values obtained for the pore size of PS samples grown by the electrochemical anodization method.

| **Sample** | **J (mA/cm^2^)** | **t (min)** | **Concentration HF - Surfactant** | **Pore size diameter (**$\emptyset$**)** |
| --- | --- | --- | --- | --- |
| m1a | 2 | 30 | [1:9] | Not appreciable |
| m1b | 2 | 60 | [1:9] | 2 nm<$\emptyset$≤50 nm |
| m1c | 2 | 120 | [1:9] | Cracks ≤25 µm |
| m2a | 4 | 30 | [1:9] | Not appreciable |
| m2b | 4 | 60 | [1:9] | Cracks ≤ 1,5 µm |
| m2c | 4 | 120 | [1:9] | Cracks ≤ 8 µm |
| m3a | 8 | 30 | [1:9] | Cracks < 4 µm |
| m3b | 8 | 60 | [1:9] | ≤ 1,7 µm |
| m3c | 8 | 120 | [1:9] | Not appreciable |
| m4b | 2 | 60 | [1:7] | 25 nm<$\emptyset$≤1,32 µm |
| m4c | 2 | 120 | [1:7] | Electropolished, Cracks < 5 µm |
| m5a | 4 | 30 | [1:7] | Cracks < 10 µm |
| m5b | 4 | 60 | [1:7] | ≤ 70 nm |
| m6b | 8 | 60 | [1:7] | < 3 µm |
| m6c | 8 | 120 | [1:7] | < 2,2 µm |
| M9Et | 100 | 1 | [1:2] | ≤ 10 µm |
| M1Et | 50 | 1 | [1:2] | Not appreciable |
| M17Et | 150 | 10 | [1:2] | ≤ 170 nm |
| M19Et | 180 | 20 | [1:2] | Cracks < 5 µm |
